# An integrated and sustainable EU health information system: national public health institutes’ needs and possible benefits

**DOI:** 10.1186/s13690-016-0171-7

**Published:** 2017-01-18

**Authors:** Petronille Bogaert, Herman Van Oyen, for BRIDGE Health

**Affiliations:** Departement of Public Health and Surveillance, Scientific Institute of Public Health, Rue Juliette Wytsmanstraat 14, 1050 Brussels, Belgium

**Keywords:** Health information, European Union, Health information system, Health monitoring, Health policy, BRIDGE Health, Stakeholder consultation, National public health institutes

## Abstract

**Background:**

Although sound data and health information are at the basis of evidence-based policy-making and research, still no single, integrated and sustainable EU-wide public health monitoring system or health information system exists.

**Main body:**

BRIDGE Health is working towards an EU health information and data generation network covering major EU health policy areas. A stakeholder consultation with national public health institutes was organised to identify the needs to strengthen the current EU health information system and to identify its possible benefits. Five key issues for improvement were identified: (1) coherence, coordination and sustainability; (2) data harmonization, collection, processing and reporting; (3) comparison and benchmarking; (4) knowledge sharing and capacity building; and (5) transferability of health information into evidence-based policy making. The vision of an improved EU health information system was formulated and the possible benefits in relation to six target groups.

**Conclusions:**

Through this consultation, BRIDGE Health has identified the continuous need to strengthen the EU health information system. A better system is about sustainability, better coordination, governance and collaboration among national health information systems and stakeholders to jointly improve, harmonise, standardise and analyse health information. More and better sharing of this comparable health data allows for more and better comparative health research, international benchmarking, national and EU-wide public health monitoring. This should be developed with the view to provide the tools to fight both common and individual challenges faced by the Members States and their politicians.

## Background

Policy-making and decision-making processes should be evidence-based and supported by adequate health information systems [1]. The best scientific evidence derived from sound data and relevant research is a prerequisite for the development of relevant public health strategies and policies and the assurance of adequate health service provisions [2, 3]. In spite of that, the European Commission (EC) and its Member States have failed to set up a holistic and integrated health information system. There is no single comprehensive EU-wide public health monitoring system or health information system that allows policy-oriented research or advice [4]. The current EU health information and data infrastructure system is fragmented and sectorial with issues of timeliness and usefulness [5]. There is still a huge area in which no health information system of comparable quality exists such as non-communicable diseases, even though chronic diseases are the main cause of death and poor quality of life in Europe [6].

Discussions on the development of an EU health information system started as early as 1994 [7]. Eight different programs were established as an initial strategy for putting in place actions on public health at European level [8]. Those included programmes on communicable diseases, cancer, rare diseases, injury prevention and drug prevention. Efforts during the subsequent health programmes have led to the current EU health surveys, European Community Health Indicators (ECHI), the communicable disease infrastructure in the EU, regulations at the Community level and many other successful projects. The harmonisation and collection of data resulting from these projects provide useful inputs to research and national and European decision makers, help to pool scarce resources and reduce the burden of health reporting at both Member State and European level. However, these projects have also demonstrated that there are significant gaps and deficiencies that need to be overcome such as diversity of health services and health information structures in Europe; fragmentation of databases and registries; health information inequality, and lack of sustainability of health information activities.

Under the lead of Eurostat, the European Statistical System provides a solid working basis for gathering and providing health data. In addition, the Organisation for Economic Cooperation and Development (OECD) and the World Health Organisation Regional Office for Europe (WHO-EUR) collect and process health information. These organisations now coordinate a selection of statistical data collections and have increased their collaboration over the years. However, in the eyes of stakeholders in the health information area, international organisations do not yet collaborate optimally [9]. Gaps and deficiencies persist. Large differences and health information inequalities can be found between Member States in both the quality and availability of health data. Additionally, the different health information areas are not systematically covered in the EU. Activities in drug control, infectious disease control, medicines, cancer and rare diseases are respectively covered by the European Monitoring Centre for Drugs and Drug Addiction, the European Centre for Disease Prevention and Control, the European Medicine Agency and the Joint Research Centre. This does not cover by far the integral area of public health and healthcare, not even the most important public health and health care challenges.

Over time, the EU institutions have continued expressing their interest and the need for a sustainable and integrated EU health information system in the EC health strategy ‘Together for Health’[10], ‘European health information – objectives and organization’[11] and the health for growth program [6]. In 2013, the Council of the EU conclusions invited the EC and Member States “to cooperate with a view to establishing a sustainable and integrated EU health information system … built on what has already been achieved through different groups and projects … exploring in particular the potential of a comprehensive health information European Research Infrastructure Consortium (ERIC) as a tool” [1]. This gave rise to the call “Towards a sustainable health and monitoring and reporting system” in the Work Programme 2014 of the Public Health Programme of community action in the field of health (2014–2020) and the set-up of the BRIDGE Health project [12].

### The BRIDGE health project

BRidging Information and Data Generation for Evidence-based Health policy and research (BRIDGE Health) is working towards an EU health information and data generation network covering major EU health policy areas by promoting the coordination and convergence of existing key projects in health information [13]. It assures knowledge transfer from past health and research frameworks in domains of population and health system monitoring, indicator development, health examination surveys, environment and health, population injury and disease registries, clinical and administrative health data collection systems and methods of health systems monitoring and evaluation. The goal of BRIDGE Health is to investigate the current situation and explore the possibilities to create an organisational entity that could take up the tasks that come with the need for strengthening the EU health information system. After investigating structural and institutional options, BRIDGE health aims to develop specific actions of such a structure and to support the transition towards it. Overall, BRIDGE health stimulates the discussion on improving the EU health information system and provides expert advice and opportunities to do this in an EU context. This paper focuses on the current EU health information system and the needs and opportunities studied through a stakeholder consultation with national public health institutes.

### Definition of EU health information system

Within this context, an EU health information system is defined *as an integrated effort to collect, process, analyse, report, communicate and use comparable health information and knowledge covering all Member States to understand the dynamics of the health of EU citizens and populations in order to support policy and decision-making, programme action, individual and public health outcomes, health system functioning, outputs and research in the European Union*. This definition is based on the WHO definition of a health information system and adapted by the BRIDGE Health partners [14].

### Stakeholder consultation with national public health institutes

BRIDGE Health has undertaken a stakeholder consultation meeting with EU national public health institutes in March 2016. The consultations aimed to identify the national public health institutes’ needs to strengthen the current EU health information system and their vision of an integrated and comprehensive EU health information system. All 28 Member States’ national public health institutes or corresponding institutes were invited to attend the meeting. A questionnaire was circulated before the meeting where participants were asked: what and if there is a need for an EU health information system, what could be the added value of such a system, and where improvements can be made in health information at EU level. During the meeting, the topics were further discussed in focus groups. The discussions were guided by moderators through a semi-structured interview. The consultation meeting was attended by 17 participants from 13 European countries. Ten responses to the questionnaires were received and the focus groups were composed of 14 participants in total.

### Needs to strengthen the current EU health information system

Five key needs to improve the current EU health information system were identified: (1) coherence, coordination and sustainability; (2) data harmonization, collection, processing and reporting; (3) comparison and benchmarking; (4) knowledge sharing and capacity building; and (5) transferability of health information into evidence-based policy making.

#### Coherence, coordination and sustainability

Currently, a variety of EU institutions, agencies and projects perform activities on health information in the EU. Representatives of national public health institutes pointed out there is a huge need for improved coordination between the various health information activities, creating synergies and sustainability. Moreover, there is no overarching governance structure that can decide on common issues such as priorities in health information, norms in data quality, etc. The tasks, roles and mandates for the different stakeholders involved in governance need to be defined. As a consequence, there is no coherent EU health information strategy having a holistic approach or transparent co-ordination. This gives rise to issues such as the many overlaps and a waste of resources, concurrent with enormous gaps between projects’ agendas, EU health (information) priorities and the scarce uptake of research results into public (health) practice and policy. A governance structure for EU health information is also needed for interdisciplinary cooperation with other policy sectors and civil societies.

#### Data harmonisation, collection, processing and reporting

Respondents repeatedly indicated there is a need for harmonisation of data definitions and indicators between countries. Systematic and sustainable governance concerning the definition and content of the indicators is needed. Also standardised methodological approaches and norms to the collection of data are required which can adapt to national infrastructures and simultaneously enable better data quality throughout the EU. Besides ensuring sustainable data collections and data availability for evidence-based public health, a governance structure at EU level is also needed, according to representatives of national public health institutes, to facilitate usage of collected data by e.g. strengthening EU health information dissemination strategies.

#### Comparison and benchmarking

Respondents to the questionnaire urged that a sound EU health information system allows Member States to have a more precise picture of the situation in their country and compare their outcomes to other Member States and regions. Member states are facing common challenges, such as demographic changes, increase of the burden of chronic diseases, increases health care costs or health inequalities. International comparisons based on selected indicators provide valuable comparative information on the extent of these challenges in Member States, as well as on measures taken to meet them. Thus, as mentioned by a respondent, “numbers and trends in my country are discussed and evaluated against the background of information from other countries. The European Core Health Indicators provide a good basis for these comparisons.” Comparing health information among EU-wide sets of health care providers, regions and countries allows health researchers to take advantage of the ‘natural experiment’ that is provided by the various types of interventions and practices that have been initiated throughout the EU. The availability and comparability of the data becomes even more essential then. Another respondent points out this would also enable comparison of data between different societies. “Through this we could really know what the magnitude of inequalities is in societies and to assess the quality and efficiency of health care system in a specific society in comparison to others.”

#### Knowledge sharing and capacity building

By sharing knowledge and expertise across borders, Member States can learn from each other to develop shared solutions and guidelines. Health determinants that operate across national boundaries can be better addressed. Member States need to pool efforts as resources are scarce and focus on improving the resilience of their health systems. Common tools and mechanisms at EU level can be identified to overcome common challenges, where strong health information and research networks foster EU–wide cooperation. A governance structure for EU health information is needed to engage in scientific exchange via structured virtual and integrated platforms. This provides an ideal platform to address health information inequalities in Member States and EU.

#### Transferability of health information and evidence-based policy making

Policymakers can only respond effectively to population and health systems’ challenges and evaluate policy measures if they have the appropriate tools and knowledge. Developing, implementing and evaluating EU actions enables members states and the European Commission to work together to effectively and efficiently develop, implement and monitor national, regional and European initiatives to achieve a higher level and more equitable distribution of health and wellbeing across the European population. A strong governance framework for EU health information would allow efficient resource allocation through better prioritization and reduced duplication of activities. Moreover, the link between health information activities (including research and development) and policy needs can be improved. Strong data systems will help to fill the data and information needed for health in all policies.

### Possible benefits of an integrated and sustainable EU health information system

National institutes of public health identified potential benefits for the setting up of an integrated and sustainable system (Table 1). Benefits in relation to six target groups were identified throughout the stakeholder consultation including decision-makers, researchers, healthcare providers, citizens, administrators or data providers, and financers. Participants to the consultation meeting also had a precise vision of an improved EU health information system. The emphasis was on the provision of relevant, reliable and comparable health indicators across the EU in a regular way. The means are by enabling safe and easy exchange of data and knowledge within the public health community. This should be made possible through the provision of a platform (1) to facilitate the use of commonly agreed indicators (ECHI) for national data collections, (2) to develop, implement and monitor national, regional and European initiatives and (3) to enable Members States and the European Commission to work closer together. The outcome of this improved EU health information system should be to objectively support effective and efficient public health policy making. An EU health information system should thereby make a substantial contribution to improving the health of the population in the EU and achieve a higher level and more equitable distribution of health and wellbeing across the population.

**Table 1:** Possible benefits for stakeholders in Member States

Decision-makers	Researchers
- Quality information for evidence-based decisions - Better preparedness - International comparison: evaluate and discuss how to tackle similar challenges - Programme evaluation - Priority setting - Organise and coordinate public health expertise and systems - Better access to existing knowledge and expertise	- EU-comparative data - Data quality - Continuous availability - Enhanced research capacity and international collaboration - Larger study populations and cohorts - Enhanced data access flow - Structured scientific exchange - Quicker results - Better access to existing knowledge and expertise
Healthcare providers	Citizens
- Data to set standards and protocols for evidence-based care and to evaluate their policies - Benchmarking i.e. learning from best practices - Better access to existing knowledge and expertise	- Improved health and wellbeing by enhanced monitoring of health risks, health status, health determinants, and the safety and quality of healthcare services - Patient reported outcomes and experiences (PROMS and PREMS) - Reduced health inequalities: promoting equitable distribution of health and wellbeing - Better access to existing knowledge and expertise
Administrators/data providers	Financers
- Reduce burden by increasing harmonisation of international data collection to reduce duplication - Assist in obligation to provide data to international sources	- Better value for money in international health information activities and health research - Optimise funds allocation

## Conclusion

Despite the acknowledged value and the many achievements in this field, the current EU health information system is still highly fragmented and lacks sustainability, coherence, and comprehensiveness. There is no comprehensive EU-wide public health monitoring system or health information system that allows policy-oriented research or advice. Through the stakeholder consultation, BRIDGE Health has identified the continuous need to strengthen the EU health information system. A better system is about sustainability, better coordination, governance and collaboration among national health information systems and stakeholders to jointly improve, harmonise, standardise and analyse health information. More and better sharing of this comparable health data allows for more and better comparative health research, international benchmarking, national and EU-wide public health monitoring. This should be developed with the view to provide the tools to fight both common and individual challenges faced by the Members States and their politicians. A better EU health information system would require a mechanism of sustainable governance, priority setting for data collection and indicator development, data analysis and common effective reporting mechanisms. All beyond the health information activities that are already undertaken by international organisations that are developing common harmonized health statistics.

## Abbreviations

BRIDGE Health: Bridging information and data generation for evidence-based health policy and research
EC: European Commission
ECHI: European Community Health Indicators
EU: European Union
OECD: Organisation for Economic Cooperation and Development
WHO-EUR: World Health Organisation Regional Office for Europe
